# Artificial Neural Networks Combined with the Principal Component Analysis for Non-Fluent Speech Recognition

**DOI:** 10.3390/s22010321

**Published:** 2022-01-01

**Authors:** Izabela Świetlicka, Wiesława Kuniszyk-Jóźkowiak, Michał Świetlicki

**Affiliations:** 1 Department of Biophysics, University of Life Sciences, Akademicka 13, 20-950 Lublin, Poland; 2 Faculty of Physical Education and Health in Biała Podlaska, Józef Piłsudski University of Physical Education in Warsaw, Akademicka 2, 21-500 Biała Podlaska, Poland; wieslawa.jozkowiak@awf.edu.pl; 3 Department of Applied Physics, Faculty of Mechanical Engineering, Lublin University of Technology, Nadbystrzycka 36, 20-618 Lublin, Poland; m.swietlicki@pollub.pl

**Keywords:** principal component analysis, stuttering, speech recognition, artificial neural networks

## Abstract

The presented paper introduces principal component analysis application for dimensionality reduction of variables describing speech signal and applicability of obtained results for the disturbed and fluent speech recognition process. A set of fluent speech signals and three speech disturbances—blocks before words starting with plosives, syllable repetitions, and sound-initial prolongations—was transformed using principal component analysis. The result was a model containing four principal components describing analysed utterances. Distances between standardised original variables and elements of the observation matrix in a new system of coordinates were calculated and then applied in the recognition process. As a classifying algorithm, the multilayer perceptron network was used. Achieved results were compared with outcomes from previous experiments where speech samples were parameterised with the Kohonen network application. The classifying network achieved overall accuracy at 76% (from 50% to 91%, depending on the dysfluency type).

## 1. Introduction

Speech, the most important means of communication, whose main aim is to send and receive messages in the linguistic form, might be disturbed or interfered with by physiological or physical factors, e.g., stuttering, becoming unintelligible for the listener. Stuttering is a break or interruption of normal speech, such as repetition, prolongation, or interjection of syllables, sounds, words, phrases, and silent pauses or blocks [1,2,3,4]. Despite a large body of research, our understanding of stuttering still lacks consensus and stuttering assessment by humans is perceived as subjective, inconsistent, time-consuming, and prone to error [5]. The main reason is an arising discrepancy among judges who do not have any unequivocally defined criteria at their disposal [6,7,8,9] or an objective automatic recognition system, which could support their judgements [10]. Pathological speech, especially stuttering, is characterised by variations in breathing, phonation, speech speed, speech rates, rhythm and pronunciation [3,11], which procure it to be much more complex. Moreover, people with speech impediments generate signals whose application in pattern recognition systems is not as effective as for clear speech [12] because a lot of information is unclear, mixed and hidden. 

Generally, pattern recognition systems consist of two main components: feature analysis and pattern classification. Most state-of-the-art speech recognition systems are based on hidden Markov models (HMMs) or artificial neural networks (ANNs), or HMM and ANN hybrids [12,13,14,15]. Neural networks play an important role both in speech [15,16,17] and speaker recognition [18,19,20,21], mainly due to the development of new neural network topologies as well as training and classification algorithms [14,22,23]. They have also been used for tasks such as classification [12,24,25] or feature extraction [26,27], isolated word recognition [28], small and large vocabulary and continuous speech recognition [29,30], as well as in disordered speech processing [7,8,12,13,31,32,33,34,35,36]. However, results achieved by recognition systems strongly depend on the input data. An adequately defined feature set could make the classification process more effective and efficient [37,38], but, in some cases, e.g., stuttering, the amount of data is so huge that further analysis is complicated, and results are difficult to interpret [39,40]. Among the wide range of methods applied in the feature extraction process, e.g., MFCC, PLP and others, principal component analysis (PCA) might be found [37,41]. The PCA method creates new features, lower in dimension, projecting the original feature vectors into a new space. This reduction is achieved by a linear transformation to a new set of variables. New variables are uncorrelated and ordered according to their importance in representing the original variables. PCA proved useful and effective by numerous applications in engineering, biology, social science, and speech processing [41,42,43,44,45]. 

The authors of the following article decided to use the PCA algorithm for feature extraction both for the disrupted and fluent speech samples. The constructed model was then used in stuttered speech recognition with the artificial neural network application.

## 2. Materials and Methods

### 2.1. The General Outline of the Experiment

**Figure 1:**
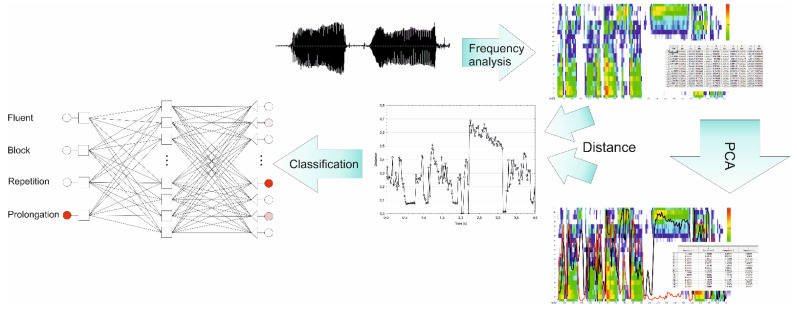
The experiment outline. The speech signal is transformed with FFT, 1/3 octave filters, and A-filter. Next, the PCA algorithm is applied. Based on the PCA model, the distances between new (PCA) and previous system of coordinates are calculated, and then the classification process with multilayer perceptron is conducted.

The study’s objective was to test the applicability of principal component analysis in disrupted signal processing and the usefulness of achieved data in the process of disfluency recognition. Therefore, the following scheme of the experiment, presented in Figure 1, was proposed. In the first step, the speech signal was transformed with Fast Fourier Transform (FFT), 21 digital 1/3-octave filters with centre frequencies from 100 to 10,000 Hz, and an A-weighting filter. Finally, the signal took a form similar to that transmitted from the inner ear to the brain.

The second stage of the experiment aimed to reduce the dimensionality of the vector describing each sample with the PCA algorithm application. To convert data matrix $X=\left[x_{ij}\right]$ achieved from frequency analysis, with dimensions [*d* × *m*], where *d* is the number of observations (171) and *m* is the number of variables (21) into a new matrix $G=\left[g_{ij}\right]$ with dimension [*d* × *l*], where *l* ≤ *m*, ***X*** needs to be standardised according to Equation (1):

(1) $$z_{ij}=\frac{x_{ij}-\bar{x}}{\sigma }$$

where *x_ij_*—original variable element, $\bar{x}$—a mean of the original variable, and *σ*—original variable standard deviation. Then, correlation matrix ***S*** could be defined as an [m × m] matrix with elements equal to correlation coefficients between variables, i.e., the product of eigenvectors ***E*** and corresponding eigenvalues **Λ** (Equation (2))

(2) $$S=E\Lambda E^{T},\;$$

where: $E=\left[\begin{array}{ccc} e_{11} & \ldots & e_{1m}\\ \vdots & \ddots & \vdots \\ e_{m1} & \ldots & e_{mm} \end{array}\right]$ and $\Lambda =\left[\begin{array}{c} \begin{array}{cc} \lambda_{1} & 0\\ 0 & \lambda_{2}\\ \vdots & \vdots \end{array}\;\begin{array}{cc} \ldots & \;0\\ \ldots & \;0\\ \ddots & \;\vdots \end{array}\\ \begin{array}{cc} \;0\; & 0 \end{array}\;\begin{array}{cc} \ldots & \;\lambda_{m} \end{array} \end{array}\right]$.

As seen (Equation (2)), ***E*** matrix columns create eigenvectors corresponding to correct eigenvalues. If we assume that we want to have only those vectors that describe the highest part of the original data variability represented by the most significant l eigenvectors corresponding to the *l* largest eigenvalues, the matrix with only *l*, instead of m columns, is received (Equation (3)):

(3) $$E_{l}=\left[e_{1},e_{2},\;\ldots ,\;e_{l}\right],$$

In the last step, the principal components matrix ***G*** was calculated by projecting original data to new dimensions determined by eigenvectors according to Equation (4):

(4) $$G=ZE_{l},$$

As a result of such an operation, matrix [*d* × *l*] was achieved, where each column contained particular principal component elements. Then, each variable from ***Z*** could be described by principal components, i.e., each element of the ***Z*** matrix can be represented as a linear combination of first *l* components. Standardised original data can be retrieved by simple data transformation (5):

(5) $$Y=(E_{l}^{T}G)+\bar{Z},\;$$

where ***Y***—original data prediction by the PCA model and $\bar{Z}$—standardized variable mean [46].

Then, distances between standardised original data matrix ***Z*** and corresponding elements *y_ij_* from the new system of coordinates were calculated according to relation (6) and used in the classification process with ANN application:

(6) $$d_{i}=\sqrt{\frac{\sum_{j=1}^{k}{\left(z_{ij}-y_{ij}\right)}^{2}}{m-l}},$$

where: *z_ij_*—element of the standardised original data set, *y_ij_*—the corresponding element of the set of observation matrix in the new system of coordinates (prediction acc. to the model), *m*—number of original variables, and *l*—number of principal components.

Calculated distances represent the residuals that allow detection of excessive distances to the model and thus map the data structure and their “shape” [46]. An observation for which the distance is equal to 0 follows the model structure, and the greater it is, the further the given observation lies from the model. Therefore, the calculated distance can be used to determine which feature the model considers as the most important and against which the entire model is built. It is a fundamental issue in constructing speech recognition models, especially concerning disfluent speech. Most of the state-of-the-art feature extraction algorithms, including Mel-frequency Cepstral Coefficients (MFCC), Linear Prediction Coefficients (LPC), Linear Prediction Cepstral Coefficients (LPCC), Discrete Wavelet Transform (DWT) or Perceptual Linear Prediction (PLP), are sensitive to noise and achieve excellent results for clear speech [35,47,48]. However, any disruption, especially prolongation, decreases their efficiency. The authors of the following article want to present a different attitude towards stuttered speech recognition, highlighting that it is not a model but the difference between the model and original data, which may indicate areas important in recognizing and classifying disturbed speech.

To test the effectiveness of the proposed solution, it was planned to make the comparison with a feature extraction method described in [31,32], where the Kohonen network was used in the process of dimensionality reduction. All the calculations and simulations were done with Statistica (TIBCO Software Inc. Palo Alto, CA, USA) application.

### 2.2. Speech Samples Preparation and Processing

One hundred and ninety-eight 4-s utterances containing three types of stuttering episodes (blocks before words starting with plosives, syllable repetitions, and sound-initial prolongations) and fluent speech were selected from the recordings of 19 people who stutter (PWS) and fluent speakers (FS), distinguished according to gender and age [31]. The recordings were made before therapy as well as during its various stages. They included two situations: reading story fragments and describing illustrations (the list of samples is placed in Supplementary Materials, Table S1). The same stories and pictures were used to record fluent samples. Patients and fluent speakers were matched according to the similarities in the signal spectrums. A female voice was recognized as equivalent to stuttering women and children, while the male voice was paired with male PWS. The detailed characterisation of the dataset is presented in Table 1.

Disfluencies were assessed in the two-stage process—firstly by four students with two years of experience at the non-fluent speech processing and next by two experts in the area of stuttering. The evaluation process aimed to select a representative sample of the particular non-fluency as far as possible without other disfluency types. The research material was recorded in an acoustic booth. The signal was transformed from an analogue to a digital form with a sampling frequency of 22,050 Hz and a sampling precision of 16 bits. Recorded samples were then analysed by the FFT 512 with a time resolution of 23 ms. An A-weighting filter and 21 digital 1/3-octave filters of centre frequencies between 100 and 10,000 Hz were used.

As the result of the analysis, a 171 × 21 matrix for each sample was achieved. The number of vectors (171) was determined according to the time resolution, while the number of their elements (21) was due to the number of filters.

### 2.3. Principal Components Analysis

The data matrix from the previous analysis was scaled to the unitary standard deviation, which means that the PCA was done on the correlation matrix. All variables were treated on an equal footing. Principal components were determined with the use of v-fold cross-validation. Individual features of the utterances (timbre, fundamental frequency, levels of formants) influenced the number of principal components, which varied among the samples from 4 to 7. To select the optimum number of variables, both the scree plots and the Kaiser–Guttman criterion were used, showing that those principal components are significant for which eigenvalues are greater than unity and explain at least 75% of the total variability. In this manner, the number of principal components was reduced to l=4 for all analysed utterances. Finally, 198 samples were transformed to a new system of coordinates, whereas each sample was represented by [171 × 4] matrix. The distances between transformed data and eigenspace were calculated based on the assumption that PCA also helps distinguish classes [40]. Received distance vectors, in dimensions [171 × 1] for each sample, were gathered in one [198 × 171] matrix and used in the classification process.

### 2.4. Kohonen Network Application

The same 198 vectors built with frequency analysis results were transformed by the Kohonen network. Since SOM (Self-organising Map, Kohonen network) can detect the most important features, it was assumed that its application provides the possibility of converting the sound signal into a character string that describes the examined utterance. Therefore, the network aim was to transform a 171 × 21 matrix into a 171 × 1 matrix, where 171 represents time points, 21 is the number of 1/3 octave filters, and 1 is the column of winning neurons at particular time points. The self-organising Kohonen network, built with 5 × 5 output neurons, trained through 100 epochs with a stable learning rate (0.1) and neighbourhood decreasing from 3 to 0, was used. As the result of the conducted analysis, the output matrix 198 × 171 consisting of neurons winning in a particular time frame was obtained and used in the classification process collaterally with the matrix received from the PCA analysis. The mentioned method was widely described in [31].

### 2.5. Recognition Process and Results Assessment

Multilayer perceptron (MLP) was trained on the data representing 55 blocks, 46 syllable repetitions, 59 sound-initial prolongations and 38 fluent utterances, divided randomly into three groups—training, validation and test, as shown in Table 2.

The network was supposed to distinguish among three disfluencies and fluent speech, so it has 171 inputs and four outputs. The number of hidden layers and neurons in each was determined based on the growth method and amounted to one hidden layer with eight neurons. The classifying network was trained with Broyden–Fletcher–Goldfarb–Shanno (BFGS) algorithm [49] for 100 epochs, with a 0.1 learning rate and constant momentum equal to 0.3. As an error function, the cross-entropy (*CE*) was applied (Equation (7)) because *CE* implementation allows interpreting output values as the probabilities of the object group membership:

(7) $$E_{CE}=-\sum_{i=1}^{N}y_{i}\left(\frac{d_{i}}{y_{i}}\right),$$

where: $y_{i}$—real output value,$\;d_{i}$—expected output value, and *N*—the number of teaching pairs input–output. Classification results were then compared with those achieved by the same classifier taught with the same parameters and working on the data received from the SOM method. Finally, selected classifiers were assessed with classification accuracy (*acc*) (Equation (8)) and overall error rate (*ε*) determined based on a testing set (Equation (9)):

(8) acc=NcNt,

(9) ε=1−acc,

where: *N_c_*—correctly classified cases from the test set and *N_t_*—the number of all test cases.

## 3. Results and Discussion

The main premise of the dimensionality reduction process is that it should be conducted to protect from losing important information, which is of the utmost importance, especially when it comes to disfluent speech recognition, where each detail could bring information needed for correct recognition or classification. Two data dimensionality reduction methods are compared: the first uses the proposed PCA transformation, while the second is based on the Kohonen network application (SOM).

### 3.1. Frequency Ranges Contribution to the PCA Model

**Figure 2:**
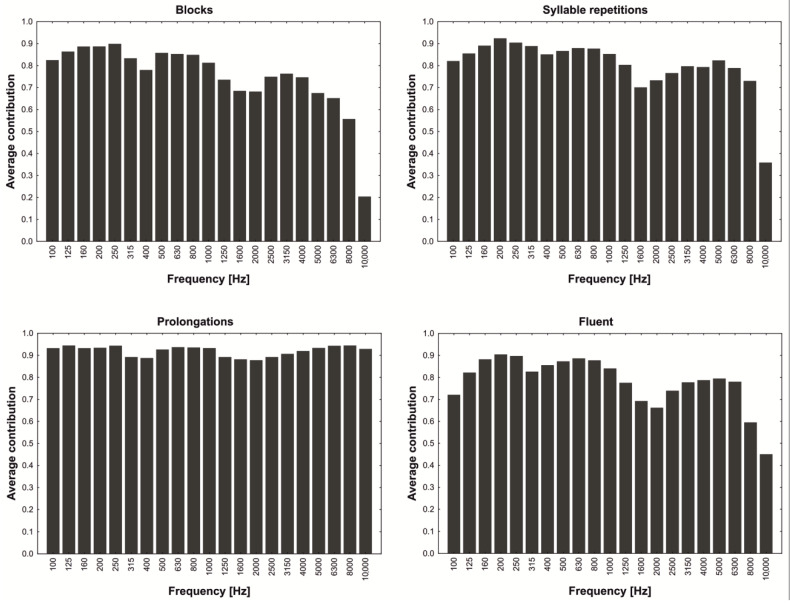
The average contribution of variables to the PCA model according to the analysed utterance type.

The number of variables used to create the PCA model amounted to 21. Due to the fact that all variables were scaled to the unitary variance, each of them had an equal chance to be represented in the model. During analysis, variables were rated according to their contribution to the model. Figure 2 presents the average contribution (*S*) of each variable in non-fluent and fluent groups calculated according to the Equation (10) and averaged in groups:

(10) S=1−SVjSVj0 ,

where: *SV_j_*—*j*th variable remainder variability and *SVj*_0_—*j*th variable variability.

The achieved contribution represents variables’ ability to model the data—a contribution close to one means that a variable was fully used in the model. As it can be seen (Figure 2), the general course of the envelope is quite similar for blocks, syllable repetitions and fluent speech. For prolongations, the chart is of a slightly different shape. Almost all frequencies prove to have the same significance (about 0.9). The main difference can be noticed in the range of frequencies from 3150 to 10,000 Hz—those frequencies seem to be much more important for prolongations than for the rest of the analysed samples. The reason for that may be that, in the Polish language, the most often prolonged sounds are sibilants and nasals, which are characterised by a concentration of energy in the range of higher frequencies [50]. Therefore, from the shapes of the plots, it can be concluded that individual components represent some parts of the frequency structure of analysed utterances.

### 3.2. The Attempt at an Interpretation of the Role of Particular Principal Components in the Description of the Speech Signal

According to PCA theory, each component explains some part of data set variability. The first of them usually describes the greatest part of it. At the same time, each further component is chosen to ensure that it will not be correlated with previous ones and will explain most of the residual variability. As a result, each additional component explains the decreasingly lower part of variability, and, consequently, consecutive eigenvalues *e_ij_* are becoming lower. The signs and values of eigenvalues *e_ij_* explain how variable *i* affects component *j*. However, it is more precise and easier to interpret so-called factor loadings *a_ij_* (Equation (11)), which also reflect the influence of particular variables ***Z*** on a given principal component ***G***. The higher the $\left|a_{ij}\right|$ value, the more significant the variable influence on building the principal component. If the analysis is conducted on the correlation matrix, it is possible to interpret the factor loadings as correlation coefficients between original data and particular principal components [51]. Such an approach makes it possible to interpret the ‘area’, which each component explains:

(11) $$a_{ij}=\sqrt{\lambda_{i}}e_{ij},$$

where: *λ_i_*—eigenvalue and *e_ij_*—eigenvector corresponding to the *i*-th eigenvalue.

**Figure 3:**
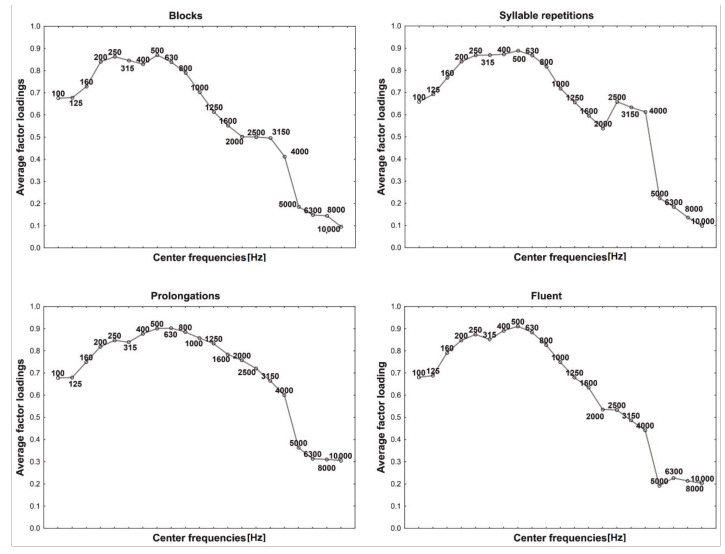
First component (G_1_) factor loadings.

**Figure 4:**
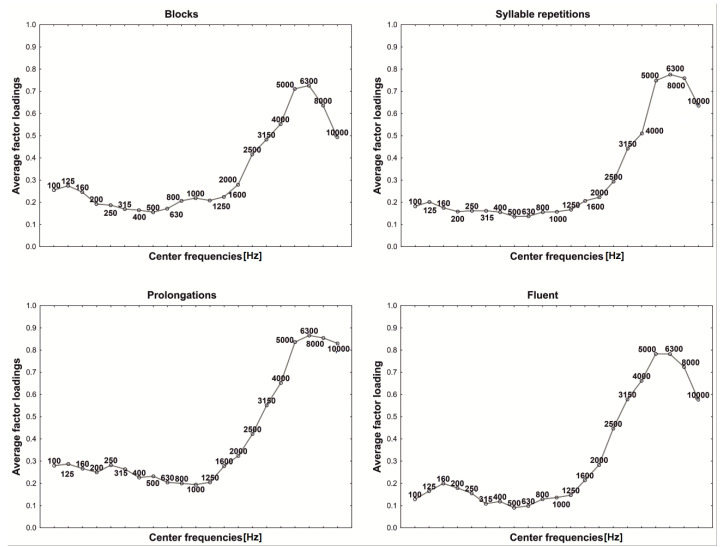
Second component (G_2_) factor loadings.

**Figure 5:**
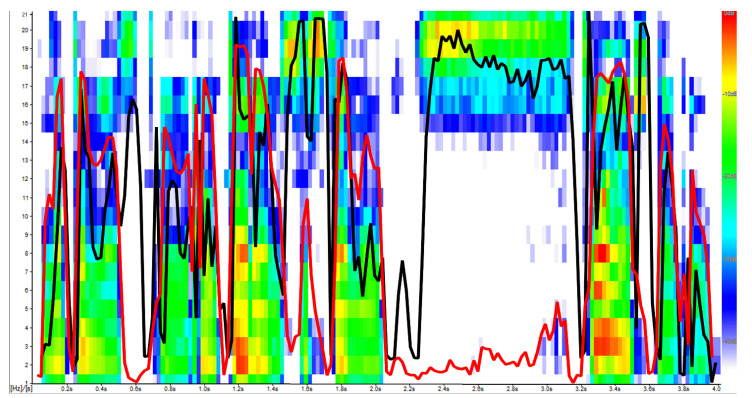
Prolongation spectrogram with G_1_ (red) and G_2_ (black) components. As can be observed, the shape of both G1 and G2 reflects the general time-frequency structure of the analysed utterance, but it is G2 which reflects higher frequencies while G1 concentrates on the lower ones instead.

**Figure 6:**
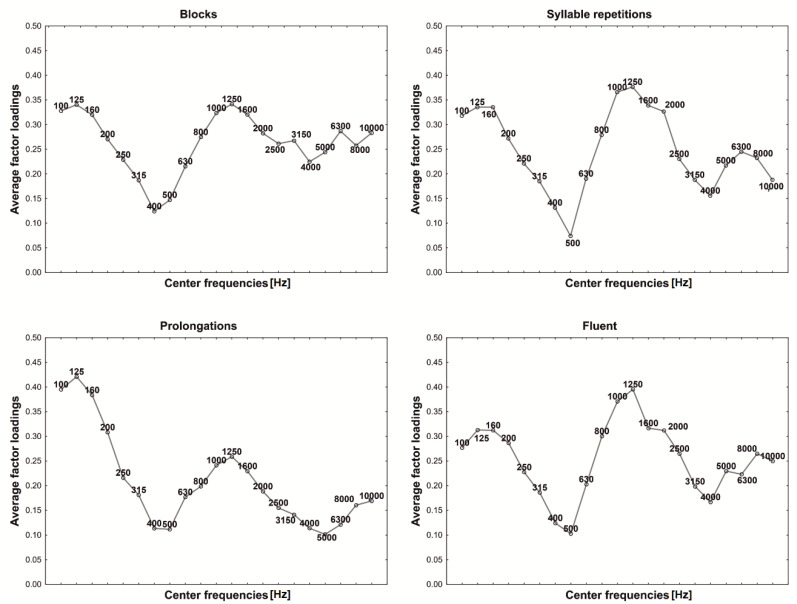
Third component (G_3_) factor loadings.

**Figure 7:**
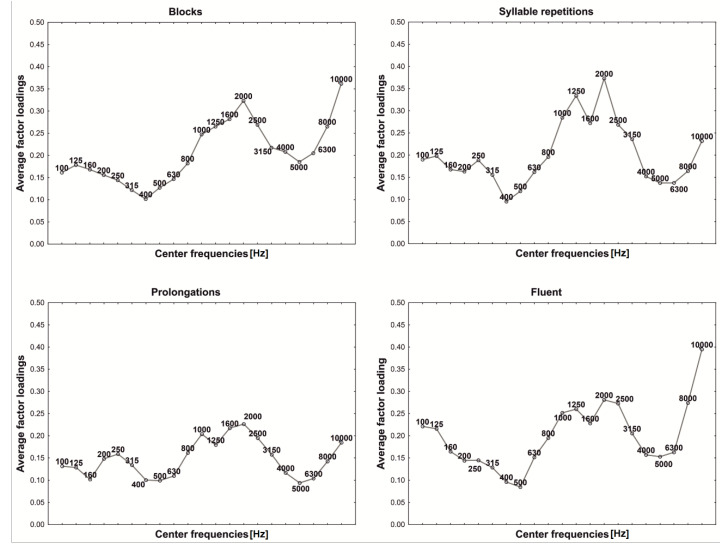
Fourth component (G_4_) factor loadings.

Trying to explain the role of each principal component, for all examined groups, the average factor loadings were calculated and illustrated in the figures, where the vertical axis gives central frequencies and the horizontal axis plots the average factor loading values (Figure 3, Figure 4, Figure 5, Figure 6 and Figure 7). For fluent and non-fluent utterances, the average factor loadings for G_1_ were the highest in the frequency range from100 to about 3000 Hz, while G_2_ was mainly constructed based on frequencies from 4000 to 10,000 Hz (Figure 3, Figure 4 and Figure 5).

Based on the obtained results, it can be concluded that the main variability of the original variables explained by the first two components divides the area of the features into lower and higher frequencies. The first component, explaining the greatest part of variability, represents low frequencies, dominant in the Polish language. In contrast, the second explains the remaining part of the variability, comprising less common, higher frequencies.

In turn, the greatest contribution to constructing the third and fourth components has particular values of frequencies, not their ranges (Figure 6 and Figure 7). This creates specific problems with interpretation, but it can be suspected that the frequencies differ within the particular utterance groups. It is, therefore, possible that those two additional components bring more details into the general image of utterance transferring properties characterising the particular kinds of disfluency or fluent speech.

In order to verify whether and which of the variables building the principal components affect the differentiation of the particular kinds of utterance, the ANOVA was applied and the Tukey RIR test for unequal groups. The results revealed that the variables included in the first component permit primarily to differentiate between fluent and non-fluent utterances (*p* < 0.05). In the case of the second component, as expected, the notable contribution of higher frequencies mainly causes prolongations to be identified (*p* < 0.05), whereas variables with the most significant contribution in building the third and fourth components permit the differentiation of utterances within the disfluencies themselves, i.e., they permit the differentiation of, e.g., a block from repetition or a prolongation from a block. However, they allow to the greatest extent to differentiate blocks (*p* = 0.001) and to the least repetitions of syllables (*p* = 0.041).

### 3.3. Distance Calculation

**Figure 8:**
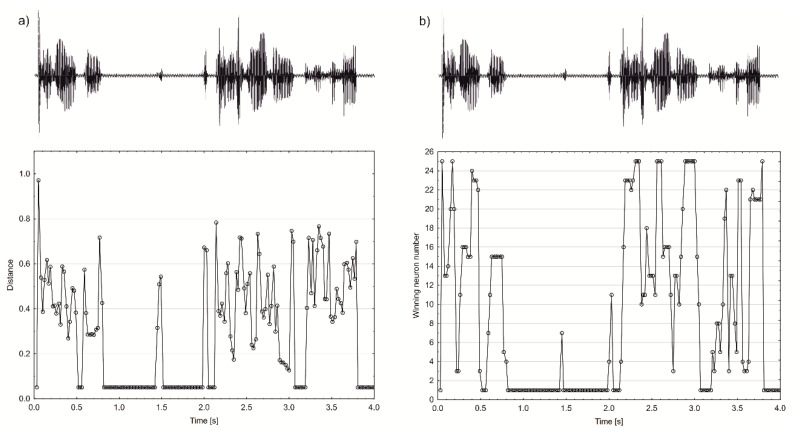
The representation of sound repetition with the PCA (**a**) and Kohonen (**b**) algorithm application.

For obtained distance values d_i_, plots of the distance-time relations were prepared (Figure 8a). The results showed that the smallest distance from the model was characteristic of silence fragments. In contrast, the greatest distances were noted at the moments of realisation of fragments with higher frequencies. The above observations suggest that the considered model is built based on moments of silence, i.e., absence of signal. At the same time, every other fragment of utterance already constitutes a divergence from the base model. As a result, the distances from the model become proportionally larger with relation to the frequency band characterising a given utterance fragment. Nevertheless, as observed in Figure 8a, both the syllabic structure and moments of silence were preserved. Considering previous conclusions, it might be put forward that PCA analysis reproduces the time-frequency domain of speech in significantly fewer dimensions.

The sample structures transformed with the PCA (Figure 8a) and Kohonen network (Figure 8b) show high similarity. However, slight differences could be noticed: the intensity of repetitions and detailed structure of fluent parts are relatively higher for the PCA method. In contrast, higher and lower frequencies seem to be separated to a greater degree for the Kohonen method.

### 3.4. Classification Results

The task of the MLP classifier was to isolate four groups from the presented data set: blocks, syllable repetitions, prolongations and fluent utterances. The results achieved by the classifier during the training process with the feature set received from the PCA method are presented in Table 3. For comparison, the classification results with the feature set created with the SOM method are also demonstrated.

As it can be seen, the same classifier trained with the use of two data sets produced results differing to a significant degree. Notably, better results were obtained for the input data resulting from PCA analysis—training, test and validation rates are about 30% higher than the SOM extraction method’s classification results. In similar research [42], where for signal dimensionality reduction, PCA was used, classifying a feedforward neural network achieved very high accuracy (close to 100%) with 5 to 8 features, while for 3, only 67.1% was gained. However, comparing achieved results to the models where authors for stuttered speech recognition used Mel-frequency cepstral coefficients (MFCC) in the process of feature extraction [39], or MFCC, PLP and FBE features [40], the accuracy ranged between 63.5% for syllable repetitions (for eight MFCC coefficients) to 98.6 % in case of interjections (for 39 MFCC coefficients). Others [52] showed that the PCA algorithm reduced the feature dimensions from 26 to 12, resulting in the same speech recognition accuracy as the conventional MFCC method without PCA, and increased speech recognition accuracy for ten feature vectors.

Taking a closer look at the detailed results and comparing the values in Table 4 and Table 5, it can be concluded that the classifier working on PCA data is more efficient and commits fewer mistakes than in the case of SOM features. Furthermore, a significantly higher degree of recognition was achieved in the group of blocks and prolongations (the higher of 14 and 18%, respectively) and of fluent utterances, where the result was improved by 71%. Only in the case of syllable repetitions did the classifier using the effects of SOM analysis prove to be more effective (difference in the range of 25%). The presented results confirm the conclusions from Tukey’s RIR test, according to which PCA analysis could be useful mainly for the differentiation between fluent utterances and prolongations, and to a lesser degree in the case of blocks.

In [31], where the Kohonen network was used for feature extraction and three MLP networks were applied to classify utterances into two non-fluent and fluent groups, the accuracy achieved 84–100 % depending on the disfluency type, which is a much better result. However, it should be taken into account that the designs of the experiments differ—in the presented research, four groups were distinguished by one neural network, while, in [31], each of the three networks distinguished only between fluent and disfluent utterances. If the feature extraction method based on the Kohonen network is considered, the tested network achieved much worse results than for the vector based on PCA (Table 4 and Table 5).

As it might be seen, the PCA algorithm enhances the previously proposed system for stuttered speech recognition and proved to be more effective. Recently, many reports have indicated the effectiveness of the algorithm’s application for feature extraction. As shown in [53], where an adaptive moment-based backpropagation algorithm of ANN (BPVAM) has been compared with PCA combined with BPVAM in the detection of Parkinson disease based on speech data, PCA application made the system to be relatively more effective. A method that hybridizes the principal component analysis (PCA) and t-statistics for feature extraction from EEG signal was proposed in [54]. Extracted features were then used by four classifiers: support vector machine (SVM), artificial neural network (ANN), linear discriminant analysis (LDA), and k-nearest neighbour (kNN), among which ANN and SVM showed the highest classification accuracy.

**Figure 9:**
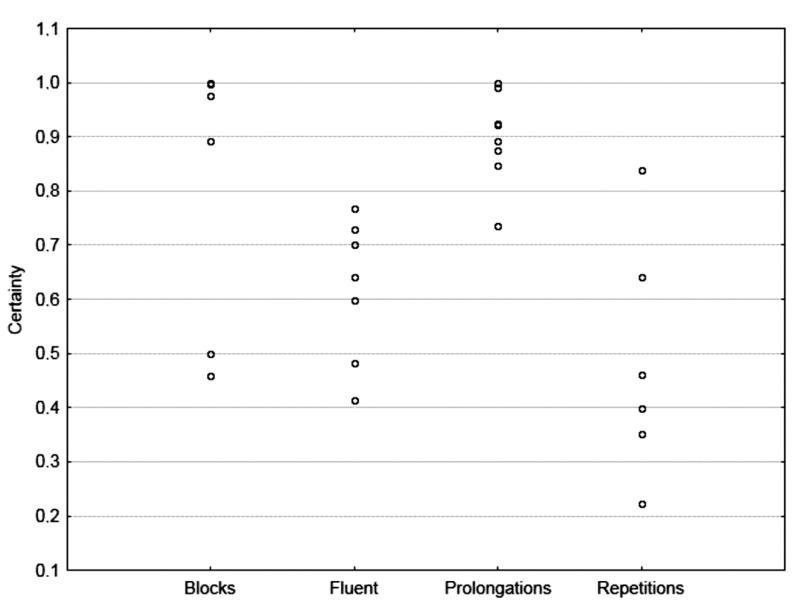
The certainty of classification concerning the fluency type.

The plot of the relation between classification certainty, understood as the output value for the classifying network and denoting the probability of the object belonging to a given class, and the type of utterance for the testing set (Figure 9) shows that prolongations are classified with the highest certainty, above 0.7, followed by blocks and fluent utterances. However, the classifier does not ensure certainty concerning particular cases from the syllable repetitions group.

While our findings provide evidence of PCA usefulness in the feature extraction process concerning stuttered speech, the presented work has some limitations which require comments. First of all, as mentioned above, not all disfluencies are pictured with the same precision, which translates into significantly worse classification results. Achieved results are probably caused by a too-small number of teaching cases within the range of this type of disfluency or incorrectness or error in describing a given kind of utterance using PCA analysis. Additionally, combining the PCA with some state-of-the-art classification algorithms, such as deep learning or Long Short-Term Memory (LSTM) and Convolutional Neural Network (CNN), which proved to be an effective tool in acoustic signal classification [55,56], might enhance the results of the proposed feature extraction method. For example, in [57], it was shown that the classifier, built with a deep residual network and bidirectional long short-term memory layers, recognised different types of stutters with almost 90% accuracy. In turn, in [58], the authors presented an algorithm based on deep learning and neural networks, which proved to be helpful to diagnose stuttered speech and during therapy sessions, which seems to indicate the high potential of the methods mentioned above.

## 4. Conclusions

The presented work introduces the method where principal component analysis and artificial neural networks were applied to support the stuttered speech recognition process. The proposed solution covers the principal component analysis application as a dimensionality reduction tool, creating a new, simplified picture of disfluent speech in the frequency domain and using ANNs as classifiers. As a result, the representation of speech samples received by frequency analysis was reduced from 21 to 4 dimensions with PCA application without a significant loss of information. Furthermore, a new sample representation by four principal components preserved the utterance time structure and reproduced silent moments. Therefore, it could be concluded that speech signal analysis with PCA application allows for receiving speech features that could constitute the basis for its recognition. The proposed method was additionally compared with the previously developed algorithm using the Kohonen network for feature extraction. Although the approach discussed showed a significant increase in accuracy, it revealed not to be a universal method for all tested stuttering types, as detecting syllable repetitions was not impressive.

The research conducted on the principal component analysis application in a non-fluent speech analysis showed that the PCA algorithm might be treated as a valuable tool in speech processing, even a disfluent one. However, more attention and tests in the area of particular speech disorder types are needed.

## Figures and Tables

**Table 1:** Characteristics of stutterers and fluent speakers regarding realised disfluency, age and gender.

	People Who Stutter			Fluent Speakers		
Disfluency Type	Number	Gender	Age Ranges (years old)	Number	Gender	Age Ranges (years old)
blocks	11	9M and 2F	10–23	8	4M and 4F	22–50
syllable repetitions	6	4M and 2F	11–23	6	4M and 2F	22–53
prolongations	7	7M	10–25	4	2M and 2F	24–51
Total	**19**	**16M and 3F**	**10–25**	**14**	**9M and 5F**	**22–53**

M—male, F—female. Cited from [31].

**Table 2:** Data distribution according to the disfluency type and affiliation to the training set.

	The Number of Samples			Total
Sample Type	Training Set	Validation Set	Test Set	
blocks	37	9	9	55
syllable repetitions	36	5	5	46
prolongations	42	5	12	59
fluent	25	10	3	38
Total	140	29	29	**198**

**Table 3:** Recognition rates (for training, validation and test set) for an MLP classifier trained with features received with PCA and SOM application.

Feature Extraction Method	Recognition Rate		
	Training	Validation	Test
PCA	92.14	72.41	75.86
SOM	59.29	55.17	51.72

**Table 4:** Classification statistics for MLP classifier calculated for the test set for PCA and SOM feature extraction methods.

	*acc*	*ε*
PCA	0.76	0.24
SOM	0.52	0.48

**Table 5:** Accuracy for examined groups of disfluencies and fluent speech calculated for the test set for PCA and SOM feature extraction methods.

	Accuracy [%]			
	Blocks	Syllable Repetitions	Prolongations	Fluent
PCA	71.43	50.00	90.91	71.43
SOM	57.14	75.00	72.73	0.00

## Data Availability

Data reported in this manuscript will be available upon request.

## Supplementary Materials

The following are available online at https://www.mdpi.com/article/10.3390/s22010321/s1, Table S1. Disfluent samples characteristics.
