# Supplementary material for: Artificial Neural Networks Combined with the Principal Component Analysis for Non-Fluent Speech Recognition
Source: Sensors (Basel). 2022 Jan 1;22(1):321. doi: 10.3390/s22010321 (PMC8749906; doi:10.3390/s22010321)
Supplement: Supplementary file 1 [file sensors-22-00321-s001.zip › sensors-1514050-supplementary.pdf]

Supplementary Material

Artificial Neural Networks Combined with the Principal Component Analysis for Non-Fluent Speech Recognition

Izabela Świetlicka [1,*], Wiesława Kuniszyk-Jóźkowiak [2] and Michał Świetlicki [3]

[1] Department of Biophysics, University of Life Sciences, Akademicka 13, 20-950 Lublin, Poland; izabela.swietlicka@up.lublin.pl
[2] Faculty of Physical Education and Health in Biała Podlaska, Józef Piłsudski University of Physical Education in Warsaw, Akademicka 2, 21-500 Biała Podlaska, Poland; wieslawa.jozkowiak@awf.edu.pl
[3] Department of Applied Physics, Faculty of Mechanical Engineering, Lublin University of Technology, Lublin, Poland; m.swietlicki@pollub.pl
* Correspondence: izabela.swietlicka@up.lublin.pl

Table S1. Disfluent samples characteristics.

No	Disfluency Type	Disfluency Duration [ms]	Time after Last Repetition [ms]	Number of Repetitions
1	b	1394	471	2
2	b	1201	390	2
3	b	1930	171	1
4	b	2928	66	2
5	b	2480	471	1
6	b	789	23	1
7	b	834	159	1
8	b	1396	273	3
9	b	466	49	1
10	b	1205	113	4
11	b	1017	13	5
12	b	803	39	6
13	b	1855	71	3
14	b	2125	70	2
15	b	2328	62	3
16	b	1053	74	6
17	b	2630	71	9
18	b	1306	117	5
19	b	2234	89	6
20	b	2217	126	7
21	b	1345	425	2
22	b	2546	27	9
23	b	1917	86	6

24	b	2446	1175	8
25	b	1645	46	5
26	b	245	66	1
27	b	696	442	1
28	b	479	318	1
29	b	424	289	1
30	b	2599	104	11
31	b	491	292	1
32	b	421	215	1
33	b	2536	479	4
34	b	903	146	1
35	b	2316	31	4
36	b	852	31	1
37	b	2135	59	1
38	b	768	102	2
39	b	1193	89	3
40	b	709	351	1
41	b	1473	786	1
42	b	2337	570	1
43	b	1596	429	2
44	b	1047	310	1
45	b	1279	464	2
46	b	934	128	3
47	b	1043	427	1
48	b	1517	63	3
49	b	830	189	3
50	b	702	38	3
51	b	2096	789	1
52	b	1034	390	1
53	b	649	41	1
54	b	2355	483	4
55	b	1690	228	2
56	sr	1094	137	1
57	sr	2620	1287	1
58	sr	1863	861	1
59	sr	1798	94	2
60	sr	525	103	1
61	sr	2764	35	2
62	sr	2640	630	1
63	sr	1717	65	2
64	sr	601	183	1
65	sr	607	231	1
66	sr	484	55	1
67	sr	1420	1041	1
68	sr	1786	84	2

69	sr	1006	471	1
70	sr	1298	273	1
71	sr	850	434	1
72	sr	1181	477	1
73	sr	975	533	1
74	sr	1042	443	1
75	sr	2244	716	1
76	sr	558	141	1
77	sr	753	224	1
78	sr	272	45	1
79	sr	891	88	2
80	sr	290	50	1
81	sr	758	123	1
82	sr	505	144	1
83	sr	516	94	2
84	sr	378	165	1
85	sr	388	47	1
86	sr	1250	69	1
87	sr	261	47	1
88	sr	1452	72	2
89	sr	1800	53	2
90	sr	637	71	1
91	sr	818	88	3
92	sr	2442	322	4
93	sr	1609	126	3
94	sr	1185	391	1
95	sr	1292	67	1
96	sr	1302	180	1
97	sr	1629	18	2
98	sr	700	73	1
99	sr	665	74	1
100	sr	911	55	2
101	sr	2051	205	5
102	sp	560		
103	sp	640		
104	sp	597		
105	sp	522		
106	sp	579		
107	sp	1639		
108	sp	587		
109	sp	2769		
110	sp	2859		
111	sp	1668		
112	sp	1939		
113	sp	577		

114	sp	1796		
115	sp	477		
116	sp	2777		
117	sp	2698		
118	sp	2941		
119	sp	2320		
120	sp	709		
121	sp	567		
122	sp	639		
123	sp	801		
124	sp	1371		
125	sp	903		
126	sp	548		
127	sp	369		
128	sp	466		
129	sp	570		
130	sp	1893		
131	sp	688		
132	sp	821		
133	sp	700		
134	sp	706		
135	sp	414		
136	sp	1289		
137	sp	707		
138	sp	816		
139	sp	358		
140	sp	1164		
141	sp	702		
142	sp	617		
143	sp	972		
144	sp	403		
145	sp	622		
146	sp	454		
147	sp	419		
148	sp	499		
149	sp	787		
150	sp	1968		
151	sp	1603		
152	sp	1288		
153	sp	1011		
154	sp	767		
155	sp	2224		
156	sp	507		
157	sp	574		
158	sp	831		

159	sp	496		
160	sp	2845		

b – blocks, sr – syllable repetitions, sp – syllable prolongations.
